# Ensuring Healthy American Indian Generations for Tomorrow through Safe and Healthy Indoor Environments

**DOI:** 10.3390/ijerph120302810

**Published:** 2015-03-04

**Authors:** Joseph A. Pacheco, Christina M. Pacheco, Charley Lewis, Chandler Williams, Charles Barnes, Lanny Rosenwasser, Won S. Choi, Christine M. Daley

**Affiliations:** 1 Center for American Indian Community Health, University of Kansas Medical Center, 3901 Rainbow Blvd. MS 1030, Kansas City, KS 66103, USA; E-Mails: cpacheco@kumc.edu (C.M.P.); clewis4@kumc.edu (C.L.); cwilliams7@kumc.edu (C.W.); wchoi@kumc.edu (W.S.C.); cdaley@kumc.edu (C.M.D.); 2 Department of Family Medicine, University of Kansas Medical Center, 3901 Rainbow Blvd., Kansas City, KS 66103, USA; 3 Department of Preventive Medicine and Public Health, University of Kansas Medical Center, 3901 Rainbow Blvd. MS 1008, Kansas City, KS 66103, USA; E-Mail: cbarnes@cmh.edu; 4 Department of Allergy, Asthma, and Immunology, Children’s Mercy Hospitals and Clinics, 2401 Gillham Rd., Kansas City, MO 64108, USA; E-Mail: lrosenwasser@cmh.edu

**Keywords:** American Indians, healthy home, home assessment, environmental

## Abstract

American Indians (AI) have the highest rate of severe physical housing problems in the U.S. (3.9%). Little information exists about the environmental hazards in AI homes. The purposes of this paper are to discuss challenges that were encountered when recruiting AI for a home-and employment-based environmental health assessments, highlight major successes, and propose recommendations for future indoor environmental health studies. The Center for American Indian Community Health (CAICH) and Children’s Mercy Hospital’s Center for Environmental Health and Allergy and Immunology Research Lab collaborated to provide educational sessions and healthy home assessments for AI. Through educational trainings, more than 240 AI were trained on the primary causes of health problems in homes. A total of 72 homes and places of employment were assessed by AI environmental health specialists. The top three categories with the most concerns observed in the homes/places of employment were allergens/dust (98%), safety/injury (89%) and chemical exposure (82%). While some information on smoking inside the home was collected, these numbers may have been underreported due to stigma. This was CAICH’s first endeavor in environmental health and although challenges arose, many more successes were achieved.

## 1. Introduction

Environmental exposures directly impact many health outcomes [1], and different strategies are necessary for reducing exposures specific to indoor environments [2,3]. Estimates predict that Americans spend roughly 90% of their time indoors, making indoor environments an important exposure site for various indoor allergens [4], chemicals, environmental tobacco smoke, volatile organic compounds (VOCs), heavy metals and unintended injuries [5]. Additionally, homes are a major predictor of health [3], with evidence showing a strong relationship between housing quality and health outcomes [5]. Sub-populations, including children, pregnant women and the elderly, are disproportionately impacted by unhealthy homes and indoor environments due to the amount of time spent indoors [3,6,7,8,9]. Low-income, racial/ethnic minority neighborhoods also carry a disproportionate burden of substandard and poor quality housing [2,3,10].

AI, defined by the United States (U.S.) Census as peoples having origins in any of the original peoples of North, Central and South America who maintain tribal affiliation and/or community attachment [11], have some of the poorest documented health outcomes of all racial/ethnic groups in the U.S. [11,12]. Additionally, AI have some of the highest rates of asthma (9.4%) compared to other racial/ethnic groups (multiracial 14.1%; Blacks, 11.2%; Whites, 7.7%; and Asians 5.2%) [12] and the highest rate of severe physical housing problems in the U.S. (3.9%), followed most closely by Blacks (2.8%) [4]. Additionally, 3% of AI live with moderate physical housing problems (malfunctioning plumbing, heating, or electrical systems, dilapidated public areas, or inadequate maintenance) [4,13]. AI also have the highest rates of smoking of any racial/ethnic group in the U.S., with rates as high as 41%, compared to 21% to 24% for Whites and Blacks, meaning children are more likely to be exposed to environmental tobacco smoke [14,15]. Due in part to housing conditions and exposure to tobacco smoke, AI children are more likely than any ethnic group to have asthma (OR = 1.82 *vs.* Whites) [16].

Despite the high proportion of AI living in unsafe and unhealthy homes, particularly AI children, there is little information in the literature about environmental hazards in AI homes. This lack of knowledge is a barrier to providing successful interventions targeted at health issues resulting from unsafe and unhealthy indoor environments. Home and place of employment assessments can aid in filling the gap of knowledge in this area. The purposes of this paper are to discuss challenges that were encountered when recruiting AI for a home-and employment-based indoor environmental health assessments, highlight major successes as the first undertaking of a project of this kind, and propose recommendations for future indoor environmental health studies.

## 2. Healthy Homes Program

### 2.1. Project Population

The State of Kansas is currently home to four federally recognized AI tribes (the Iowa Tribe of Kansas and Nebraska, the Kickapoo Tribe in Kansas, the Prairie Band Potawatomi Nation, and Sac and Fox Nation of Missouri in Kansas and Nebraska), one federally operated tribal university (Haskell Indian Nations University), and many rural and urban AI who do not live on reservations. According to the American Community Survey 5-Year Estimates there were 62,579 AI (inclusive of people selecting multiple races) living in the State of Kansas between 2009 and 2013 [17]. In the State of Kansas, 19.3% of AI are under the age of 18 and 4.9% are over the age of 65, meaning almost 1/4 of AI in the State of Kansas [18] are more susceptible to hazardous indoor environmental exposures and housing related risks [7,8,9]. In the State of Kansas, 69% of AI live in single detached homes [19].

While the State of Missouri is not currently home to any federally recognized AI tribes, historically several AI tribes did inhabit the land currently known as the State of Missouri. However, most AI in this region were forcibly removed under the Indian Removal Act of 1830 [20]. Today, the State of Missouri is home to 78,309 rural and urban AI (inclusive of people selecting multiple races) [17]. Additionally, 13.4% of AI in the State of Missouri are under the age of 18 and 6.5% are over the age of 65, meaning roughly 1/5 of AI in the State of Missouri [18] are at greater risk of exposure to hazardous indoor environments and housing related risks [7,8,9]. In the State of Missouri, 76% of AI live in single detached homes [19].

### 2.2. Addressing Disparities through Collaboration

The Center for American Indian Community Health (CAICH) at the University of Kansas Medical Center in Kansas City, KS, was created in 2010 to reduce health disparities among AI in the Heartland. CAICH is a National Institute on Minority Health and Health Disparities funded Center of Excellence. Its objectives are to: (1) bring a talented, interdisciplinary group of researchers together to foster innovative translational research on health disparities among AI; (2) enhance the training of new researchers, particularly AI researchers; and (3) strengthen our innovative partnership with the regional AI community. In an effort to address additional environmental health disparities faced by AI, an area not previously explored by CAICH, we partnered with the Children’s Mercy Hospital’s Center for Environmental Health (CMH-CEH) and Allergy and Immunology Research Lab (CMH-AIRL) to launch a project that (1) raised awareness about environmental health; (2) educated community members, providers, and facilities workers about how to achieve safe and healthy indoor environments; and (3) improved home and working environments of many AI in Kansas and Missouri.

The mission of CMH-CEH is to improve and advocate for the health of people with environmentally-triggered illnesses. CMH-CEH is a nationally recognized center of excellence in children’s environmental health research, education, consulting, and analytical services and is a national leader in home environmental assessment practices. However, the AI community has been noticeably missing from previous assessments, interventions, and trainings provided by CMH-CEH; the collaboration between CAICH and CMH-CEH facilitated the expansion of both centers’ work. In an effort to address the indoor environmental health needs of the AI community, this collaboration provided educational trainings and healthy home assessments to AI community members. CMH-CEH was responsible for training AI environmental health specialists from CAICH on how to conduct the home assessments as well as how to do the community trainings. CMH-AIRL was responsible for the analysis and interpretation of all dust, air and suspect mold samples.

### 2.3. Community Trainings

We have conducted numerous educational training sessions throughout Kansas and Missouri in an effort to inform AI on how to make their homes safe and healthy environments. The trainings were adapted from the National Center for Healthy Housing’s Essentials for Healthy Homes Practitioners Training Course [21]. The trainings were modified to make them more applicable to the AI communities in KS and MO. The modifications were based on feedback from our community advisory boards (CABs) to address concerns relevant to AI in this region. Pictures were updated to depict current housing of AI and actual home issues of concern. These modifications were well received by our CABs and other AI, because they were more relatable to their current homes.

The training components included information about lead-based paint, radon, mold, and pests [21]. Trainings addressed root causes of health problems in homes and connected them to “*seven principles of healthy housing: keep it dry; keep it clean; keep it pest-free; keep it ventilated; keep it safe, keep it contaminant-free; and keep it maintained*” [21]. At the completion of the trainings, participants had a better understanding of how to make their homes healthy. Over the course of two years, we were able to train more than 240 AI.

### 2.4. Recruitment for Home Assessments

Recruitment for the environmental home assessments was conducted in multiple ways. Our first method of recruitment was through the community educational trainings described above. At the conclusion of the trainings, participants were encouraged to sign up for free environmental home assessments. Second, flyers were distributed to different AI community centers and tribal offices throughout Kansas and Missouri. At health fairs and pow wows, information booths were set up to inform AI about home health and safety and to recruit for the environmental home assessments. Other recruitment strategies included advertising via word of mouth and through AI specific list serves. In addition to face-to-face recruitment, we used social media outlets such as Facebook^©^ and Twitter^©^. However, because we did not track the method of recruitment when enrolling participants, it is unclear which method was most successful.

This service was tailored for homes; therefore, recruitment was geared solely towards homeowners and renters. We did not actively recruit for the assessment of places of employment. However, when different tribes and AI organizations learned about the service being offered, the demand was great. In response to this community demand, we modified the assessments for places of employment so that we could further meet the needs of the community.

### 2.5. Eligibility and Enrollment for Home/Place of Employment Assessments

Eligibility and enrollment for assessments were conducted by CAICH staff. Enrollment in the environmental home assessments was open to anyone who self-identified as AI and resided in Kansas or Missouri. This included multi-racial/multi-ethnic households. Participation was open to anyone who lived on or off a reservation as well as those living in single family or multifamily homes, regardless of owner/rental status. All participants were required to fill out a questionnaire prior to assessment. Sample questions from the questionnaire can be found in Table 1. The questionnaire gave the environmental health specialists additional information about the occupants’ health symptoms, as well as basic home and demographic information. Additionally, the questionnaire allowed the occupants to share information about their home from their perspective.

**Table 1:** Healthy homes intake questions.

**Health Symptoms** Does anyone in your household have any of the following symptoms when at home, and then seem to get better when (s)he leaves? ○ Headaches ○ Itchy, watery, or burning eyes ○ Breathing problems, coughing, or shortness of breath Do the symptoms get better when away from certain rooms in the home? Do symptoms get worse at night, or on the weekends? Does anyone in the household cough, wheeze, have chest tightness, or feel short of breath year round?
**Home Assessment** Can you see dust or dirt on your furniture, walls, ceiling, and curtains? Do you vacuum less than once a week? Does moisture regularly build-up on your windows or walls? Has water entered your home through the roof, windows, or plumbing leaks? Does your home have mold growing anywhere other than the shower?
**Indoor Air Quality** Does anyone smoke inside your home? Is a gas stove, kerosene, or oil heater used without ventilation in your home? Are household chemicals or sprays used regularly for household cleaning? Are there any activities in your home that generate odors, gases, or strong fumes?

Due to multiple requests, we conducted environmental assessments of buildings in which AI were employed. Intake questionnaires were not required for place of employment assessments. However, before conducting these assessments, we verified that at least 30% of employees were AI. We included this stipulation to ensure that the program was benefiting the AI community, the main focus of our mission. All employers who requested a building assessment received one. Employers were asked to list the number of AI employees working in the buildings, as well as identify any environmental issues they had. This took place through a short phone or in-person consultation.

### 2.6. Home/Place of Employment Assessments

Participant homes/places of employment were assessed by AI environmental health specialists from CAICH and the first five assessments were overseen by CMH-CEH staff. All environmental health specialists were trained by attending the National Center for Healthy Housing’s Essentials for Healthy Homes Practitioners Training Course [21].

Initially two levels of assessments were offered; level 1 and level 2. Level 1 assessments included a detailed visual evaluation of the environmental conditions inside and outside of the home and were scored using a standard set of criteria [22]. Assessments also included a look at the overall structural and mechanical systems of the home [22]. The number of rooms assessed varied and was dependent on the homeowners’ levels of comfort. Overall the number of rooms assessed at homes varied between three and ten rooms. In addition to all of the data collected, pictures were taken to visually show participants the issues of concern in their homes. Level 1 assessments were limited to examining moisture control, chemical exposure and safety and injury prevention [23]. On occasion limited samples were collected, most notably when suspect mold was identified.

Level 2 assessments encompassed all aspects of level 1 assessments and considered five general problem categories including air quality, allergens and dust, moisture control, chemical exposure and safety and injury prevention [22,23,24]. Level 2 assessments also included the collection of designated on-site samples [23]. Specific analytical parameters included measurements of temperature, humidity, carbon dioxide, carbon monoxide, airborne particulates and dust borne allergens [23,24]. Dust borne allergens that were analyzed included cat, dog, dust mite, mouse, roach, *Alternaria alternata*, *Aspergillus fumigatus*, *Cladosporium herbarum* and *Penicillium chrysogenum* [23,24]. Outdoor samples of pollen and spores were collected at each home/place of employment for comparison to indoor spore levels [23,24]. This information was considered when an air quality maintenance concern arose [23,24]. All places of employment received a level 2 assessment.

Hard copies of educational materials about indoor environmental hazards and safer cleaning were provided during assessments. CAICH environmental health specialists provided one-on-one in-home education in conjunction with the assessments. All data collected are stored in a database maintained by CAICH. After the datasets were created, all names, addresses and personal health information were removed. This database allows us to better understand the current state of housing in the AI communities in Kansas and Missouri.

After an assessment was completed, regardless of the level, the information gathered from a home or place of employment was compiled into a Healthy Home or Healthy Workplace Assessment Report. The report was divided into sections and a room-by-room summary was created. The summary was scored and color-coded based on the three-point level of concern scale for the general problem categories. In all cases the higher the score, the better the quality of the indoor health in a room. In addition to the report, families and employers received a Healthy Home or Healthy Workplace “*Intervention Action Plan*” that provided a specific list of actions to take to reduce asthma triggers, air toxins and radon, as well as recommended interventions specific for the particular home or place of employment. These reports were delivered to the home owners/employers within 2 weeks.

In an effort to reduce any bias in this study, the homes/places of employment were evaluated by at least one of six AI environmental health specialists. The AI environmental health specialist used the same multi-component checklist developed by CMH-CEH. Individual assessors routinely conducted home assessments in groups to synchronize methods and results. CAICH assessed a total of 72 homes and places of employment, 26 level 1 homes, 26 level 2 homes, and 20 places of employment.

### 2.7. Healthy Home Kits

To aid homeowners with maintaining a safe and healthy home, healthy home kits were given to participants at the completion of the home assessments. Healthy Home Kits included a culturally-tailored book for AI entitled, “*Help Yourself to a Healthy Home*” [25] provided by Alabama Cooperative Extension System. This book is an educational tool and reinforces the teaching that occurs during the assessment. More importantly the kits included a smoke detector, a carbon monoxide detector and a fire extinguisher. The vast majority of homes were missing either one or all of these things which decreased a home’s score during the assessments. All participants were educated on proper placement and use of the included items. Several homes requested multiple smoke detectors; these were provided when requested. Employers did not receive Healthy Home Kits, but did receive detailed reports as discussed above.

## 3. Major Findings

A total of 72 homes and places of employment were assessed. Participant homes were similar to other homes in area. Housing, in the areas assessed (urban, rural and reservation), is typical for the Midwestern U.S. Most homes were single-family homes, with a basement and forced air heating and cooling. Other homes were multi-dwelling units, mostly apartment buildings or duplexes. The age of homes varied widely and did not seem to be a factor in the assessment outcomes. Maintenance issues were found in all housing types regardless of home age. The places of employment that were assessed were also similar to other places of employment in the area. They varied substantially in size, age, and type (e.g., office building, community center, school, *etc.*).

**Figure 1:**
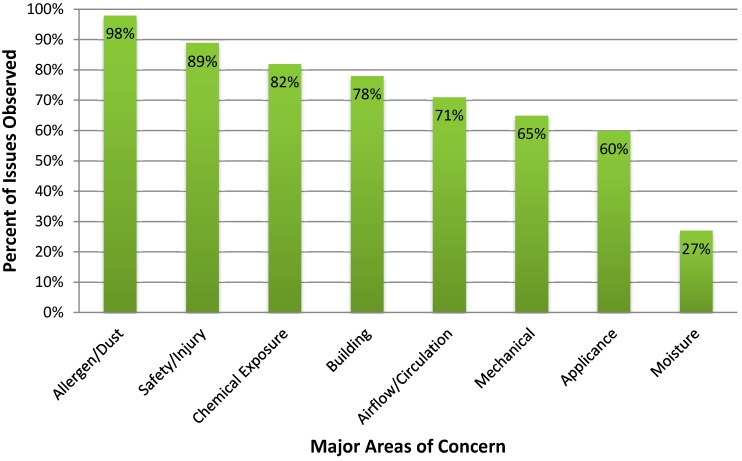
Percentage of homes/places of employment with issues observed during health home assessments by category of concern.

The percentages of homes/places of employment with issues of concern that were identified are summarized in Figure 1 by major category of concern. With regards to allergens and dust, issues were noted in 98% of the assessments. The most common issues included visible dust and the presence of clutter. In the category of safety and injury, problems in 89% of assessments included the absence of carbon monoxide detectors and/or smoke detectors or smoke detectors without a source of power. Environmental health specialists identified concerns with chemical exposure in 82% of assessments; most of these concerns included the use of air fresheners, candles and/or incense, all of which contribute to the amount of VOCs to which a person is exposed. Building/structural issues, which included flaking paint on the outside of homes/places of employment, missing or damaged downspouts, and/or missing or damaged splash blocks for guttering, were noted in 78% of assessments. Air flow and circulation issues were observed in 71% of all assessments, with the most common issues being blocked or missing supply and return vents. In 65% of all assessments, mechanical issues were observed. The most common issue in this category was related to furnace air filters (correct size/type and replacement). Environmental health specialists found issues with appliances in 60% of assessments. These issues were most commonly attributed to the lack of external exhaust systems for stoves. The least observed issues (27%) were in the category of moisture. The issues observed in this category were primarily due to reported and observed visible moisture stains and visible suspect mold.

The major respiratory illnesses reported in the homes were seasonal allergies and asthma. With education, the vast majority of home issues identified were repairable. For example, homeowners were educated about how often to change furnace filters. This small action can substantially improve air quality in the home. Some moisture issues were resolvable by simple repairs to guttering, such as including splash blocks and downspout extensions to redirect water away from the foundation. Homeowners and employers were responsible for all remediation because the grant did not cover those services. Upon follow-up with the homeowners and employers, we learned that many major issues were addressed.

## 4. Challenges

Although many AI expressed an interest in having their homes assessed, we faced many recruitment challenges. Some prospective participants expressed concerns about privacy, stating that the home assessments felt intrusive. On several occasions, one head of the household wanted the home assessment, while the other did not. Scheduling also proved to be a challenge. Many participants did not have the ability to take off work for the assessments, which were typically done during the workday. Other participants were not interested in evening or weekend assessments due to other commitments. The length of the level 2 assessments, which was 1.5 to 2 h long, was also problematic for some participants. Others did not like the idea of having “strangers” in their home for that length of time. One of the most challenging barriers to overcome was a homeowner’s feeling of embarrassment about his or her living situation. It was not uncommon for homeowners to request that no pictures be taken as a part of the assessment. Another common concern expressed by homeowners was a fear of being reported to the Environmental Protection Agency or Housing and Urban Development due to the condition of their living environment. We also received requests that pictures not be sent to *Hoarders^©^*, a cable television series that depicts dramatic stories about extreme hoarding. None of the homes we encountered had hoarding issues. In fact, most homeowners cleaned prior to our arrival, which was problematic. The assessments were most beneficial when the home was in its natural state.

In some homes, home hazards such as flooded basements, made it too dangerous to assess certain parts of homes. Other hazards included unsafe structures, exposed electrical wiring, and extensive mold problems. Upon encountering these hazards, homeowners were given and asked to sign a “Notice of Hazardous Condition”, which stated the reason why a particular room could not be assessed. Additional challenges were related to equipment malfunctions or dead batteries. Learning the new equipment and calibrating the machines was challenging for the new environmental health specialists.

## 5. Successes

This was CAICH’s first endeavor in addressing environmental health; therefore, partnering with CMH-CEH and CMH-AIRL gave this work clout. While the AI community recognizes CAICH as a vehicle for addressing health disparities such as smoking, cancer screening and obesity, it was unknown in the area of environmental health, particularly indoor environmental assessments. CMH-CEH and CMH-AIRL are nationally renowned for their healthy homes work and respected in the region, but unknown in the regional AI community. CMH’s name recognition is far reaching for its work with children. This collaboration allowed both entities to expand their work into new areas.

Another major success of this program was the resulting number of AI environmental health specialists, trained both formally and in the field. Seven AI faculty, staff and Master of Public Health students attended the National Center for Healthy Housing’s Essentials for Healthy Homes Practitioners Training Course [21]. Four additional AI CAICH staff members and interns received field training from formally trained staff during home and building assessments. Field training entailed hands-on learning and shadowing of formally trained staff. Field trained staff assisted but did not lead home and building assessments. In addition to the seven formally trained AI environmental health specialists, more than 240 AI community members received educational training on the root causes of health problems in homes through the seven principles of healthy housing [21].

We heard many anecdotal stories about AI community members feeling that the assessments made a difference in their homes and workplaces. One woman visited CAICH for the sole purpose of relaying that she felt her life was saved when a CAICH AI environmental health specialist discovered a clogged flue pipe in her furnace that would have caused carbon monoxide to build up in her home. Additionally, gas leaks were discovered in multiple homes of which homeowners were not aware. Stories such as these circulated throughout the AI community, resulting in continued interest in home/place of employment assessments.

## 6. Conclusions

### 6.1. Future Directions

The long-term success of this project rests on our ability to show that healthy indoor environments are achievable, affordable and advantageous [26,27]. This can be achieved through a three-pronged approach, including education, service and research. Through continued education, community members are learning simple and inexpensive changes to their living and working environments can have a lasting impact on their health and the safety of those environments. CAICH continues to offer healthy homes and place of employment assessments free of charge because there is a continued interest in this service. Future interventions should address the cost-benefit ratio of home improvements and specific health outcomes [27], such as asthma and injury prevention (both high in the AI population). These types of studies need to be conducted to provide concrete evidence of the benefits of healthy indoor environments.

### 6.2. Recommendations

Future studies can address ways to overcome the challenges faced during this project. For example, the challenge related to refusal rates of home assessments due to privacy concerns could be addressed through a study testing the feasibility of training homeowners/renters to do the environmental assessments on their own homes under the guidance of an AI environmental specialist. This type of study would require minimal invasion of privacy, a major concern of prospective participants, potentially resulting in higher participation rates. With homeowners/renters doing the data collection and AI environmental specialists still conducting the analysis and education, participants would still reap the benefits of the assessment.

Another potential study could couple a healthy home assessment with a smoking cessation program. For more than 10 years, one of the major areas of intervention at CAICH has been culturally-tailored smoking cessation [28]. A two-pronged approach such as this could be an ideal way to improve the overall health of all members in AI homes. The home assessment, education, and feedback would improve the physical environment of the home, while the smoking cessation component would improve the health of all home occupants by reducing second- and third-hand smoke exposure in the home, thereby impacting home occupants with allergies, asthma, and other upper respiratory diseases/illnesses.
